# Explaining burnout and the intention to leave the profession among health professionals – a cross-sectional study in a hospital setting in Switzerland

**DOI:** 10.1186/s12913-018-3556-1

**Published:** 2018-10-19

**Authors:** Oliver Hämmig

**Affiliations:** Epidemiology, Biostatistics and Prevention Institute, University of Zurich, Hirschengraben 84, 8001 Zurich, Switzerland

**Keywords:** Burnout, Intention to leave the profession, Health professionals, Physicians, Nurses, Switzerland

## Abstract

**Background:**

Burnout and the intention to leave the profession are frequently studied outcomes in healthcare settings that have not been investigated together and across different health professions before. This study aimed to examine work-related explanatory factors or predictors of burnout and the intention to leave the profession among health professionals in general, and nurses and physicians in particular.

**Methods:**

Cross-sectional survey data of 1840 employees of six public hospitals and rehabilitation clinics recorded in 2015/16 in German-speaking Switzerland were used. Multiple logistic and stepwise linear regression analyses were performed to estimate the relative risks (odds ratios) and standardized effects (beta coefficients) of different workloads and work-related stressors on these outcomes and to study any possible mediation between them.

**Results:**

On average, one in twelve health professionals showed increased burnout symptoms and every sixth one thought frequently of leaving the profession. Temporal, physical, emotional and mental workloads and job stresses were strongly and positively associated with burnout symptoms and thoughts of leaving the profession. However, the relative risks of increased burnout symptoms and frequent thoughts of leaving the profession were highest in the case of effort-reward and work-life imbalances. In fact, these two work-related stress measures partly or even largely mediated the relationships between exposures (workloads, job stresses) and outcomes and were found to be the strongest predictors of all. Whereas a work-life imbalance most strongly predicted burnout symptoms among health professionals (β = .35), and particularly physicians (β = .48), an effort-reward imbalance most strongly predicted thoughts of leaving the profession (β = .31–36). A substantial part of the variance was explained in the fully specified regression models across both major health professions and both outcomes. However, explained variance was most pronounced for burnout symptoms of physicians (43.3%) and for frequent thoughts of leaving the profession among nurses and midwives (28.7%).

**Conclusions:**

Reducing workload and job stress, and particularly reward frustration at work, as well as the difficulties in combining work and private lives among health professionals, may help to prevent them from developing burnout and/or leaving the profession and consequently also to reduce turnover, early retirement, career endings and understaffing in healthcare settings.

## Background

It is well known and has been repeatedly reported that healthcare professionals, and particularly hospital staff, face numerous hazards, precarious working conditions, high workloads and job stresses such as long and irregular working hours, physical burdens, emotional pressures, social or role conflicts, understaffing and many more. Nurses and hospital physicians in particular experience high levels of work stress as a result [1–3].

Individuals use various ways or strategies to respond to or cope with high workloads and chronic job stress. American psychologists Richard S. Lazarus and Susan Folkman in their famous and most often cited cognitive stress theory have distinguished between problem- and emotion-focused coping of stress [4]. In accordance with this theory it seems obvious and plausible that there are in principle two possible ways of coping in reaction to job stress, apart from solving the problem or rather modifying the stressful working conditions: regulation of emotions (e.g. dissociation and emotional withdrawal) or elimination of the stressor (e.g. quitting the job or leaving the profession). One way is to remain exposed to the workloads and occupational stresses and suffer from emotional and physical exhaustion at some point, and then “cool down” and distance oneself emotionally from the patients to retain one’s job functionality [1], or to “burn out”, get sick and temporarily lose one’s ability to work. Another adaptive strategy is to avoid or reduce prolonged work stress by changing the job or the organisation, or – if this does not help and solve the problem – by leaving the profession. It is not without reason that the burnout risks and turnover rates and intentions of physicians and nurses are among the most frequently reported challenges and studied outcomes in healthcare and hospital settings. Both stress reactions, burnout and leaving the organisation or profession, pose major challenges to the healthcare system.

In fact, it has long been recognized that burnout as “a consequence of continued exposure to stressful events related to work” [5] is a common occupational disease in the healthcare professions, and that turnover rates among nurses pose a challenge to healthcare systems worldwide due to staff shortages and resulting poor patient outcomes. Accordingly, burnout and the intention to leave the organisation or the profession, or at least patient care, in response to constant work stress are frequently studied among nurses and/or hospital physicians [1, 6–23].

However, most of these studies are focused either on burnout or on the intention to leave the profession (or direct patient care) and/or on only one profession or specialty, mostly nurses or physicians. Numerous and diverse work factors and stress measures were used and studied as predictors of these outcomes, but these have not been consistent. Some studies have investigated the effort-reward imbalance as a prominent job stress model and a predictor of burnout [15, 17, 21, 22] and intention to leave the profession [6, 18]. Others considered work-life/family conflict, interference or imbalance as a major explanatory factor for burnout [8, 15] or the intention to leave the profession [7, 11, 12, 24].

**Fig. 1:**
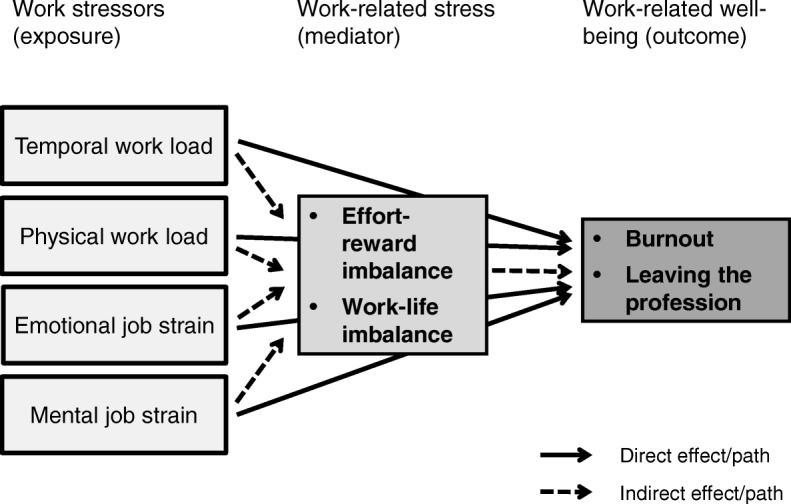
Explanatory model for the prediction of burnout und intention to leave the profession

Burnout and the intention to leave the profession have scarcely been studied together so far, and particularly not for both major health professions (nurses and physicians) simultaneously and under consideration of different work stressors and work-related stress measures. This study therefore sought to examine the relationships between four major work stressors and two prominent and most highly relevant outcomes in healthcare settings (see Fig. 1). This was done for both nurses and hospital physicians and under consideration and the assumed mediation of two identified work-related stress models or measures [15, 25], namely effort-reward imbalance and work-life imbalance.

The study aimed to answer the following research questions:

- Which work factors and particularly job stressors are most strongly associated with burnout?
- Do these work factors or stressors effect burnout more directly or indirectly, and are they mediated by effort-reward and/or work-life imbalance?
- Can the same contributing factors, predictive effects and direct and indirect paths be observed for the intention to leave the profession as the other outcome under study?
- Can any differences regarding these relationships and effects between the two major health professions be observed?

## Methods

### Data and study sample

Cross-sectional survey data recorded between summer 2015 and spring 2016 among the workforces of four public hospitals and two rehabilitation clinics in German-speaking Switzerland, including a university hospital, a cantonal hospital and a district hospital, were used for this study. The participation by hospital employees was voluntary and anonymous. The overall participation or return rate of the full sample postal survey was just over 41%, ranging from 36 to 49% depending on the hospital. The written questionnaire contained exactly 100 questions (single items) or groups of questions (scales) on “Work and Health in the Hospital”. Pre-tests have shown that it took about half an hour on average to complete the questionnaire.

A total of 1840 hospital employees, including 1441 health professionals, were interviewed, i.e. completed and returned the questionnaire. More than 85% of all participants and almost 88% of the participating health professionals were women, with a female share of more than 94% among caregivers and nurses (including midwives) and almost 64% among physicians. The participants were mostly highly educated (66%) and below 45 years of age (58%).

### Measures

#### Work stressors

Measures of four different aspects of workloads and job stresses were used as exposure variables and predictors of burnout and intention to leave the profession, namely temporal and physical workloads and emotional and mental job stresses.

- *Temporal workload* was measured by the self-reported number of extra hours worked in a standard week, ranging from 0 (no voluntary or required overtime at all) over 1–2, 3–5 and 6–10 extra hours to more than 10 extra hours per week.
- *Physical workload* was assessed by asking participants for the amount of time (the whole time, three quarters of the time, half of the time, one quarter of the time or never/almost never) they spend at work a) in painful or tiring positions (poor posture), b) carrying or moving persons, c) carrying or moving heavy loads, d) standing and e) with uniform hand or arm movements.
- *Emotional job stress* was measured by the sum score of a 5-item scale taken from the German version of the Copenhagen Psychosocial Questionnaire (COPSOQ) and with questions such as ‘Does your work put you in emotionally disturbing situations?’ (response categories: 4 = always, 3 = often, 2 = sometimes, 1 = seldom, 0 = hardly ever/never) or by asking ‘Do you get emotionally involved in your work?’ and ‘Does your work require that you hide your feelings?’ (response categories: 0 = to a very small extent, 1 = to a small extent, 2 = somewhat, 3 = to a large extent, 4 = to a very large extent).
- *Finally, mental job stress* was assessed with the sum score of five 4-point Likert scaled items selected from the 6-item subscale on over-commitment [26], also used in the COPSOQ. Items used were reports or statements of being unable to sleep at night after having left something unfinished at work, being unable to switch off from work when getting home or having a troubled mind due to work problems when waking up etc. (response categories: 0 = strongly disagree, 1 = disagree, 2 = agree, 3 = strongly agree). These items measure “the long arm of the job” and stressful work rather than excessive work engagement or over-commitment to the job as a personal characteristic or personality trait.

#### Work-related stress

Stress at work or related to work in general was assessed by two well established and validated measures of reward frustration or gratification crises at work (effort-reward imbalance) and role conflict and compatibility problems between work and private life (work-life imbalance). The effort-reward imbalance was originally conceptualized and operationalized by Siegrist and colleagues as an important and prominent stress model [26, 27] and was measured accordingly by the ERI ratio calculated from the two dimensions and the 10-item and 6-item subscales of “effort” and “reward”. The work-life imbalance was assessed with the 5-item work-privacy conflict scale used in the COPSOQ, a translated and adapted German version of the work-family conflict (WFC) subscale of Netemeyer et al. [28]. The scale includes the following items:

1. The demands of my work interfere with my home and family life.
2. The amount of time my job takes up makes it difficult to fulfill family or other private responsibilities.
3. Things I want to do at home do not get done because of the demands my job puts on me.
4. My job produces strain that makes it difficult to fulfill my family or private duties.
5. Due to work-related duties, I have to make changes to my plans for family activities.

#### Burnout

The risk of burnout was measured by the six-item personal burnout subscale of the Copenhagen Burnout Inventory (CBI), developed by Kristensen et al. [29] and Borritz et al. [30], as the most important dimension and only CBI subscale used in the standard German version of the COPSOQ. Participants were asked about the frequency of feeling tired (item 1) and drained (item 5), feeling weak and vulnerable to diseases (item 6), being physically and emotionally exhausted (items 2 und 3) and thinking ‘I can’t go on any longer’ (item 4). The response scale ranged from ‘never’ (score 0) to ‘always’ (score 4). An aggregated sum score of 16 up to the maximum of 24 was considered as constituting an increased risk of burnout.

#### Intention to leave the profession

Participants were asked not only about actually considering a job change but also and even more interestingly about having thought of leaving the profession during the previous 12 months. The question included response categories from 1 (‘never’) to 5 (‘daily’). This single-item measure was used in the famous European NEXT (Nurses’ Early Exit) study and was again taken from the standard German version of the COPSOQ.

### Analyses

First, descriptive statistics and particularly the relative frequencies of all exposure, mediator and outcome variables were calculated for the entire study population (hospital employees) as well as for the caregivers and nurses, the physicians and all health professionals separately.

Second, bivariate associations between exposure variables (work stressors) and mediator variables (stress measures) on the one hand and outcome variables (burnout, intention to leave the profession) on the other were analyzed. Logistic regression analyses were then carried out and odds ratios for the more and most highly exposed persons were calculated as proxies for their relative risks of increased burnout symptoms and frequent thoughts of leaving the profession. All studied associations were adjusted for sex, age and education. Additionally, the analyses were stratified for the two major health professions (nurses, physicians), all health professionals and all hospital staff.

Third, multivariate analyses or more specific multiple linear regression analyses were performed and standardized beta coefficients were calculated to estimate and compare the individual and independent effects of all predictors, to test for partial or full mediation in the relationship between exposure and outcome variables and to assess the explained variance (R squared) of the outcome variables. Again, these analyses were carried out for the full study sample and additionally stratified for three subsamples (nurses, physicians, all health professionals).

Since linear and mediated associations and unidirectional and dose-response relationships were postulated (see Fig. 1) or implicitly expected and no bidirectional or hidden structures or unobservable constructs (latent variables) were assumed and had to be studied and tested, multivariate logistic and linear regression analyses have been chosen as the most appropriate statistical methods for this study. Explorative statistical methods like factor or cluster analyses, simple bivariate methods like correlation analyses or sophisticated multivariate methods like Structural Equation Modeling (SEM) would have been inadequate or insufficient or overdone in one way or another.

## Results

### Descriptive statistics

Descriptive statistics have clearly shown the expected high temporal workload of physicians and the well-known high physical workload of caregivers and nurses (see Table 1). More than one third of physicians reported regular overtime of six or more hours per week compared to only 3% among caregivers and nurses and 8% among all hospital staff (including physicians). In contrast, caregivers and nurses showed a high or very high physical workload in 50% of the cases whereas this proportion was only 13% among physicians. Descriptive results also revealed the comparably (very) high emotional job stress of health professionals in total (59%) and particularly of physicians (70%), as well as the (very) high mental job stress of physicians (46%) compared to all hospital employees (33%). As a consequence, slightly or strongly increased stress levels were found for caregivers and nurses in terms of a (very) high effort-reward imbalance (71% vs. 64% among all hospital employees) and for physicians in terms of a (very) high work-life imbalance (68% vs. 33% among all hospital employees). However, no significantly increased burnout symptoms or thoughts of leaving the profession were found for caregivers and nurses and only an increased proportion of physicians with an elevated burnout risk (13% vs. 8% among all hospital employees) became evident (see Table 1). As regards the differences between the two major health professions, physicians showed a significantly higher prevalence rate of being at increased risk of burnout compared to nurses and midwives (13% vs. 7%), whereas nurses showed a clearly higher proportion frequently thinking of leaving the profession (19% vs. 14%). In total, one in twelve of all studied health professionals showed an increased burnout risk and every sixth person frequently considered leaving the profession.

**Table 1:** Workloads and job stresses, work-related stress measures and well-being among health professionals in particular and hospital employees in general

	Caregivers & nurses (incl. midwives)	Physicians	All health professionals	Total hospital employees
	*N* = 882	*N* = 235	*N* = 1441	*N* = 1840
Temporal workload				
No regular overtime	40.0%	19.1%	35.3%	37.5%
1–2 long hours/week	42.7%	22.6%	39.4%	37.0%
3–5 long hours/week	14.6%	24.8%	16.9%	17.5%
6+ long hours/week	2.7%	33.5%	8.4%	8.0%
Physical workload				
Low (0–1)	7.9%	29.7%	15.3%	17.8%
Medium (2–5)	42.6%	57.2%	47.1%	46.6%
High (6–10)	39.1%	12.2%	30.3%	28.6%
Very high (11–20)	10.4%	0.9%	7.3%	6.9%
Emotional job stress				
Low (0–5)	5.0%	1.8%	6.8%	12.6%
Medium (6–10)	35.7%	27.9%	34.1%	35.5%
High (11–15)	54.7%	64.6%	54.4%	47.6%
Very high (16–20)	4.6%	5.8%	4.7%	4.3%
Mental job stress				
Low (0–5)	27.9%	15.4%	24.8%	26.5%
Medium (6–7)	42.1%	38.2%	41.2%	40.1%
High (8–9)	23.4%	34.6%	26.3%	25.7%
Very high (10–15)	6.7%	11.8%	7.6%	7.7%
Effort-reward imbalance				
(Very) low (≤0.8)	7.4%	11.7%	10.3%	12.5%
Moderate (> 0.8–1.0)	22.2%	23.4%	23.0%	23.9%
High (> 1.0–1.5)	56.0%	53.7%	53.5%	51.1%
Very high (> 1.5)	14.5%	11.2%	13.3%	12.6%
Work-life imbalance				
Low (0–5)	29.4%	7.9%	28.7%	35.2%
Medium (6–10)	36.8%	23.7%	33.9%	32.1%
High (11–15)	27.2%	45.2%	28.6%	25.4%
Very high (16–20)	6.6%	23.2%	8.8%	7.3%
Burnout symptoms				
(Very) few (0–11)	70.0%	65.1%	68.7%	69.6%
Some (12–15)	23.3%	22.0%	22.9%	22.2%
Many (16–24)	6.7%	12.9%	8.4%	8.2%
Thoughts of leaving the profession				
Never (5)	47.0%	54.7%	49.4%	51.1%
Several times per year (4)	34.4%	31.0%	33.9%	32.8%
Several times per month to daily (1–3)	18.5%	14.2%	16.7%	16.1%

### Bivariate analyses

Logistic regression analyses revealed strong associations between all workloads and job stresses with the two health- and work-related outcomes (see Table 2). With few exceptions, clear dose-response relationships were found between exposures and outcomes. The higher the workload and job stress, the higher did the relative risk (adjusted odds ratio) of burnout and leaving the profession turn out to be. Associations and gradients were particularly strong between mental job stress on the one hand and increased burnout symptoms and frequent thoughts of leaving the profession on the other. Even stronger associations emerged between potential mediators or stress measures, namely effort-reward and work-life imbalance, and the outcomes of increased burnout symptoms and frequent thoughts of leaving the profession. The relative risks of burnout and leaving the profession turned out to be between 15 and almost 100 times higher for the most exposed or stressed compared to the least exposed persons. Such strong associations and almost consistently clear dose-response relationships are at least an indication of causation according to Hill’s criteria for causality [31].

**Table 2:** Associations of workloads, job stresses and work-related stressors with burnout and intention to leave the profession among hospital employees

	Increased burnout symptoms (16–24)			Frequent thoughts of leaving the profession (several times per month to daily)		
	%	aOR^a)^	95% CI	%	aOR^a)^	95% CI
Population at risk / affected	8.2			16.1		
Temporal workload						
No regular overtime	4.0	1		12.4	1	
1–2 long hours/week	9.1	2.47	1.53–3.97	19.3	1.70	1.25–2.31
3–5 long hours/week	12.8	3.77	2.22–6.39	17.1	1.63	1.11–2.39
6+ long hours/week	13.9	4.10	2.10–8.01	17.2	1.71	1.01–2.88
Number of cases in model		1629			1649	
Physical workload						
Low (0–1)	5.0	1		12.0	1	
Medium (2–5)	5.5	1.18	0.64–2.16	11.9	0.96	0.64–1.46
High (6–10)	11.5	2.78	1.49–5.17	21.8	1.90	1.23–2.91
Very high (11–20)	22.6	6.57	3.20–13.46	32.5	3.21	1.85–5.55
Number of cases in model		1629			1649	
Emotional job stress						
Low (0–5)	4.5	1		8.1	1	
Medium (6–10)	3.2	0.79	0.36–1.73	10.2	1.35	0.77–2.35
High (11–15)	11.4	3.12	1.56–6.24	20.6	3.32	1.96–5.63
Very high (16–20)	22.4	7.59	3.16–18.23	36.4	8.67	4.32–17.41
Number of cases in model		1706			1725	
Mental job stress						
Low (0–5)	1.9	1		6.6	1	
Medium (6–7)	5.0	2.71	1.28–5.73	13.0	2.27	1.47–3.50
High (7–9)	12.7	7.57	3.68–15.56	23.0	4.58	2.97–7.07
Very high (10–15)	31.9	25.39	11.84–54.46	39.9	10.30	6.16–17.21
Number of cases in model		1722			1740	
Effort-reward imbalance						
(Very) low (≤0.8)	0.5	1		3.4	1	
Moderate (> 0.8–1.0)	2.8	6.68	0.85–52.27	5.2	1.58	0.65–3.85
High (> 1.0–1.5)	7.6	20.00	2.75–145.6	16.7	6.54	3.00–14.29
Very high (> 1.5)	28.6	97.69	13.29–717.9	46.2	30.06	13.34–67.73
Number of cases in model		1620			1637	
Work-life imbalance						
Low (0–5)	1.9	1		6.5	1	
Medium (6–10)	3.9	2.42	1.16–5.07	11.7	2.00	1.32–3.04
High (11–15)	14.7	10.43	5.35–20.35	25.5	5.46	3.67–8.13
Very high (16–20)	33.6	32.64	15.74–67.69	45.8	14.57	8.87–23.93
Number of cases in model		1728			1747	

^a)^Odds ratios adjusted for sex, age and education

In sum, temporal and physical workloads, emotional and mental job stresses, and stress measures such as effort-reward and work-life imbalance have all proven to be strong risk factors for both burnout and intention to leave the profession.

### Multivariate analyses

Finally, stratified multiple linear regression analyses have shown that work stressors – with the exception of temporal workload – are significant and strong predictors of burnout (see Table 3), even when controlled for one another and similarly for the two major health professions, all health professionals and all hospital staff. However, these effects turned out to be partly or even completely (physicians) indirect and mediated by stress as measured by effort-reward and work-life imbalance. With beta coefficients from .35 to .48 between the four studied strata or samples, work-life imbalance consistently turned out to be the strongest predictor by far, as shown in Table 3. The four considered predictors (work stressors) and the two additionally included mediators (stress measures) together explained an impressively large proportion of the variance of burnout symptoms among caregivers and nurses (40.0%) and physicians (43.3%).

**Table 3:** Explaining burnout symptoms among hospital employees – results of stepwise multiple linear regression analyses (aggregated and stratified)

Dependent or outcome variable: Burnout symptoms (CBI score 0–24)	Caregivers & nurses (incl. midwives)		Physicians		All health professionals		Total hospital employees	
	*N* = 882		*N* = 235		*N* = 1441		*N* = 1840	
	Beta coeff. (β)		Beta coeff. (β)		Beta coeff. (β)		Beta coeff. (β)	
	Step 1	Step 2	Step 1	Step 2	Step 1	Step 2	Step 1	Step 2
Independent or exposure variables:								
Temporal workload (number of overtime hours per week 0–10+)	n.s.	n.s.	n.s.	−.13*	n.s.	−.09***	n.s.	−.07**
Physical workload (sum score 0–20)	.20***	.12***	.16*	n.s.	.15***	.07**	.17***	.09***
Emotional job stress (sum score 0–20)	.26***	.13***	.23***	n.s.	.22***	.09**	.17***	n.s.
Mental job stress (sum score 0–15)	.25***	.16***	.28***	n.s.	.31***	.20***	.34***	.23***
Intervening or mediating variables:								
Effort-reward imbalance (ERI ratio)	–	.08*	–	.17*	–	.14***	–	.12***
Work-life imbalance (sum score 0–20)	–	.35***	–	.48***	–	.35***	–	.35***
Control variables:								
Sex (male)	n.s.	n.s.	n.s.	n.s.	−.05*	−.06*	n.s.	−.05*
Age (< 25, 25–34, 35–44, 45–54, 55+)	−.13***	n.s.	n.s.	n.s.	−.13***	−.09***	−.13***	−.10***
Educational level (low, medium, high, very high)	–	–	–	–	n.s.	n.s.	n.s.	−.05*
**Adjusted R square**	.300	.400	.228	.433	.276	.383	.278	.376
No. cases in model	763	708	204	187	1242	1157	1551	1438

*p ≤ .05; **p < .01; ***p < .001; n.s. = not significant (*p* > .05)

Table 4 shows a similar pattern for the frequency of thoughts of leaving the profession as the outcome variable. Again, the predictive effects of work stressors were partly or completely mediated by the two stress measures of effort-reward and work-life imbalance. But in contrast to the number of burnout symptoms as the outcome variable, all exposure and mediating variables turned out to predict thoughts of leaving the profession somewhat less strongly (see Table 4). And this time, effort-reward imbalance rather than work-life imbalance emerged as the strongest predictor in the whole study population and the three subsamples (β = .31–.36). Accordingly, in the fully specified regression models, R squared as a measure of the explained variance was lower and ranged between 22.1% (physicians) and 28.7% (caregivers and nurses).

**Table 4:** Explaining thoughts of leaving the profession among hospital employees – results of stepwise multiple linear regression analyses (aggregated and stratified)

Dependent or outcome variable: Thinking of leaving the profession (ordinal scale from 1 ‘never’ to 5 ‘daily’)	Caregivers & nurses (incl. midwives)		Physicians		All health professionals		Total hospital employees	
	*N* = 882		*N* = 235		*N* = 1441		*N* = 1840	
	Beta coeff. (β)		Beta coeff. (β)		Beta coeff. (β)		Beta coeff. (β)	
	Step 1	Step 2	Step 1	Step 2	Step 1	Step 2	Step 1	Step 2
Independent or exposure variables:								
Temporal workload (number of overtime hours per week 0–10+)	n.s.	−.09*	n.s.	n.s.	n.s.	−.10***	n.s.	−.11***
Physical workload (sum score 0–20)	.12***	n.s.	.15*	n.s.	.12***	n.s.	.11***	n.s.
Emotional stress (sum score 0–20)	.21***	n.s.	n.s.	n.s.	.17***	n.s.	.18***	n.s.
Mental stress (sum score 0–15)	.18***	.08*	.29***	.19*	.21***	.10***	.21***	.09***
Intervening or mediating variables:								
Effort-reward imbalance (ERI ratio)	–	.31***	–	.36***	–	.34***	–	.34***
Work-life imbalance (sum score 0–20)	–	.25***	–	n.s.	–	.20***	–	.19***
Control variables:								
Sex (male)	n.s.	n.s.	n.s.	n.s.	n.s.	n.s.	n.s.	n.s.
Age (< 25, 25–34, 35–44, 45–54, 55+)	−.13***	−.09**	n.s.	n.s.	−.10***	−.11***	−.11***	−.11***
Educational level (low, medium, high, very high)	–	–	–	–	n.s.	n.s.	−.06*	−.06*
**Adjusted R square**	.166	.287	.110	.221	.143	.262	.140	.251
No. cases in model	776	715	204	187	1260	1168	1573	1450

*p ≤ .05; **p < .01; ***p < .001; n.s. = not significant (*p* > .05)

## Discussion

### Main findings

This study was performed as a consequence of a lack of research, particularly in Switzerland, on the common predictors of both burnout and intention to leave the profession, two major outcomes and challenges in healthcare. Physical, emotional and particularly mental workloads and job stresses were found to be significantly, positively and strongly associated with both outcomes under study. However, these negative effects turned out to be at least partly indirect, i.e. to be partly or even completely mediated by effort-reward and/or work-life imbalance, two recognized stress measures. Interestingly, reward frustration or occupational gratification crises (effort-reward imbalance) turned out to be most predictive for the intention to leave the profession, whereas difficulties with reconciling work and private life (work-life imbalance) emerged as by far the strongest predictor for burnout. The prevalence rates of these outcomes in the study population averaged 8% for increased burnout symptoms and 16% for frequent thoughts of leaving the profession. These two outcomes in both major health professions could be explained to a fairly large extent by the work stressors and work-related stress models considered (burnout: 40–43%, intention to leave the profession: 22–29%).

The results of the study are largely in line with findings from other studies, although hardly any other studies focused on both outcomes and both health professions simultaneously.

One of the very few generally comparable studies of the prevalence and correlates of burnout among physicians and nurses in a hospital setting in Belgium also found a particularly strong association between work-home interference and emotional exhaustion (as one of three burnout dimensions), which in turn was strongly related to turnover intention [8]. However, in the Belgian study sample 17% of the physicians and 12% of the nurses were at risk of burnout, whereas in the present Swiss study 13% of the physicians and 7% of the nurses showed an increased risk of burnout. Independently of such different levels, which may be attributed to different measures or questions, the prevalence rates in both studies were significantly higher among physicians than among nurses. This particular finding is supported by another Belgian study [32], but is not consistent with a study among nurses and physicians in a general hospital in central Italy [33].

Largely consistent with the findings of the present study, previous studies found similar risk factors or predictors of nurses’ and/or physicians’ intention to leave the profession or direct patient care, namely frequent overtime hours, high workload and poor compatibility of profession and family [12], long working hours and work-family conflict [11], time-related workload and work-life interference [7] or high effort-reward imbalance and job stress [6]. But the proportions of physicians or nurses intending to leave the profession or direct patient care vary significantly across studies and countries [11, 14]. For example, a recently published cross-sectional study that focused on intention to leave the profession among hospital nurses in Brazil showed that 22% of these Brazilian nurses often thought about giving up nursing [6] compared to nearly 19% among the nurses and midwives in the present Swiss study. A prospective cohort study among nurses in Sweden during their first five years after graduation reported a similar proportion of 18% after five years in practice who strongly intended to leave the profession [13]. In contrast, a large-scale cross-sectional and multinational European study among hospital nurses revealed an average rate of 9% of all 23,159 nurses across ten European countries and 6% of the Swiss subsample who intended to leave the profession [14]. This difference in relative frequency might be explained by using a yes/no-question about nurses’ intention to leave the hospital or the profession in the near future as was done in the European study, instead of just asking about how often they had thought about leaving the profession in the recent past as measured in the Brazilian, Swedish or the present Swiss studies. In fact, and at least among physicians, prospectively stated intentions to leave are usually less frequent than appears retrospectively [11]. Another reason for this could be that the European study used nationally representative samples, whereas the Brazilian and Swiss country studies used non-representative samples of hospitals and nurses.

In sum, the predictive effects studied and found for different work stressors and work-related stress models or measures are supported in detail but not across the board by previous studies. Thus in contrast to other studies, the temporal workload (overtime hours) did not turn out to be a risk factor for burnout or intention to leave the profession in this study. Emotional and in particular mental job stresses, which were found to be strong risk factors for the outcomes in this study, were not considered at all in any other study. The following findings of this study have not or scarcely been reported and published in previous studies: burnout symptoms were significantly more prevalent among physicians than among nurses, whereas the intention to leave the profession is more common among nurses than among physicians. And while work-life imbalance among health professionals surprisingly turned out to be a much stronger predictor of burnout risk than effort-reward imbalance, in contrast the latter emerged as a stronger predictor of the intention to leave the profession.

### Strengths and limitations

This study differs and stands out from many other studies in the following aspects:

- The study was not focused on a single health profession or an individual (health) outcome, as is usually the case, but was integrative as regards the study population, the outcomes under study and the work factors and stress models that were included. Nurses and physicians were considered together. Emotional and mental job stresses were supplemented by temporal and physical workloads and jointly taken into account as possible predictors. Both effort-reward imbalance and work-life imbalance were included simultaneously. And burnout symptoms were supplemented by thoughts of leaving the profession as a second major outcome.
- Sample size and numbers of cases of the two main health professions (nurses, physicians) were sufficiently large to allow for stratified and multivariate association analyses simultaneously, i.e. for comparisons between different health professions or occupational groups and for adjustments for different control variables and possible confounders and/or mediators.
- The use of mostly well established and validated measures and (sub)scales in the written questionnaire broadly insured the validity and reliability of the study findings.

Besides these strengths, the study also has some limitations:

- Due to its cross-sectional design, causal conclusions cannot be drawn, even though there was repeatedly talk of effects, predictors or dose-response relationships.
- Since the participating hospitals and rehab clinics were self-selected and hence not randomly selected, and the return rate of the questionnaire-based survey was rather low, the study sample is not representative for hospital staff or health professionals in German-speaking Switzerland. A selection bias due to a systematic self-exclusion of the stressed, heavily loaded and dissatisfied employees cannot be ruled out. This may possibly lead to an underestimation of the true burden of stress and disease and particularly the prevalence of burnout or the intention to change the profession among hospital staffs or certain health professions. Therefore the findings can only be generalized to a limited extent, and the prevalence rates must be treated with caution.

However, the strong associations and clear gradients which were consistently found are at least an indication for causation beyond simple association. And there is no reason to believe that participants (hospitals) and respondents (employees) differ significantly and in particular systematically from non-participants or non-responders and, hence, that the prevalence rates and especially the associations between exposures (or predictors) and outcomes would be very different in other hospitals or for other hospital staff.

## Conclusion

The inability to balance, integrate or reconcile job and family or career and personal life among health professionals obviously goes along with exhaustion rather than thoughts of leaving the profession. In contrast, being insufficiently rewarded for the effort spent at work seems to be the main reason for the intention to leave. There are ways of preventing health professionals, and particularly nurses and physicians, from getting sick and/or quitting the job, or rather leaving the profession and direct healthcare. This requires a reduction not only of their physical workloads or emotional and mental job stresses but in particular the avoidance or at least reduction of their work stress in both forms of work-life and effort-reward imbalance. The temporal workload was the only work factor of all those studied that seems not to play the expected role as a risk factor, so that reducing overtime or long hours is not a promising strategy.

## Abbreviation

CBI: Copenhagen Burnout Inventory
COPSOQ: Copenhagen Psychosocial Questionnaire
